# A Modified Surgical Method Combined with Blepharoplasty Design for Treatment of Xanthelasma Palpebrarum

**DOI:** 10.1155/2020/4803168

**Published:** 2020-11-30

**Authors:** Eun Jeong Choi, Tea Min Oh, Hyun Ho Han

**Affiliations:** Department of Plastic Surgery, Asan Medical Center, University of Ulsan College of Medicine, Seoul, Republic of Korea

## Abstract

**Purpose:**

Xanthelasma palpebrarum manifests as a yellowish placoid on the medial aspect of the upper eyelids, often in middle and old age. Aggravated lateral hooding of the eyelid might cause a deformity with conventional surgery, which appears to be more deficient on the medial side with excess hooding of the lateral skin. The authors suggest a novel surgical technique to solve this problem and reconstruct the defect appropriately.

**Methods:**

From July 2017 to December 2018, our method of combining excision with blepharoplasty was performed on 8 patients, consisting of 15 lesions on the upper eyelid and 6 lesions on the lower lid. Lesion removal incorporating blepharoplasty incision was done. After resection, the orbicularis oculi musculocutaneous flap was widely elevated extending through the whole upper eyelid with the lateral flap first along with fat maneuver. The flap was advanced into the defect, with even distribution of tension, after lower flap fixation to the required height of the eyelid fold. Taping was done with a hydrocolloid bandage and kept for 2 weeks.

**Results:**

The wounds were healed primarily, and no cases of recurrence, lagophthalmos, hypertrophic scar, pigmentation, or remarkable deformity were noted. All patients were satisfied, and the functional outcomes were excellent. Two patients had trivial complications specific to our method, that of triple fold and neo-Mongolian fold, which were simply revised later.

**Conclusions:**

This modality overcame the drawbacks of eyelid deformity observed in previous surgical methods, giving excellent results without any critical complications.

## 1. Introduction

Xanthelasma palpebrarum is a fibrohistiocytic tumor, in the category of stromal tumors, occurring in the eyelids [1]. It is a subcutaneous lesion that manifests as one or more yellowish placoid lesions, appearing frequently in the medial aspect of the eyelids or medial canthal areas. Xanthelasma is the most common type of cutaneous xanthoma, with an estimated prevalence ranging from 1 to 4 percent in the general population [2]. It can be seen both in hyperlipidemic individuals, who have primary hyperlipemia or secondary hyperlipemia, and normolipemic individuals [3]. It is more common in females than in males and is usually seen in middle-aged and older adults, especially between the age of 35 and 55 years [4]. Even though it is benign, the lesion mostly causes aesthetic concerns, with its position attracting attention and the possibility of enlargement; hence, patients want it removed. Systemic evaluation is needed to confirm the causative etiology and correct it by drug therapy or lifestyle modification [5]. Previous surgical treatment modalities included a simple excision, uncapping [6], microsurgical inverted peeling [7], skin graft [8], and modified blepharoplasty incision incorporating turnover flap [9], switch flap [6], or hinged flap [10].

**Figure 1:**
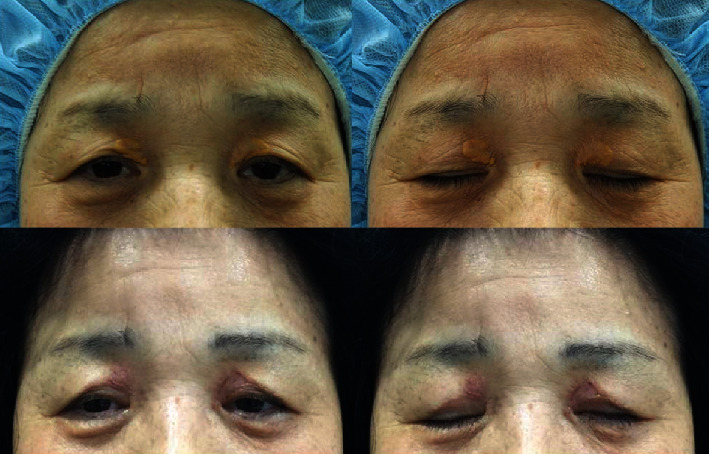
Simple excision of the medial lesion can induce deformity displaying lack of skin on medial upper eyelid and hooding on the lateral side.

The majority of patients with xanthelasma are middle aged, having lateral hooding and ptotic appearance of the upper eyelid. Simple excision of the medial skin encompassing the lesion might cause a deformity more deficient on the medial side and more hooding with excess laxity of lateral skin. When a skin graft is additionally used to cover the wide range of defects, the contracture following grafting can make it even worse (Figure 1).

In such a situation, using lateral redundant skin to cover the defect after removing the xanthelasma on the medial side can not only reduce the lateral hooding but also cover the medial defect. Compared to previous surgical techniques, the advancement and details of this technique are different [8–11]. Our technique included the orbicularis oculi muscle musculocutaneous flap elevation, separating the retroorbicularis oculi fat (ROOF) and dissecting widely from the lower eyebrow to lateral canthal area so that it can have the potential to handle the extensive defect easily. The authors suggest this novel surgical technique to solve the problems concerning the effective extraction of xanthelasma.

## 2. Patients and Methods

From July 2017 to March 2019, the resection of xanthelasma palpebrarum combined with blepharoplasty incision was performed, with coverage of the resultant defect using orbicularis oculi muscle musculocutaneous flap. This study was approved by the Asan Medical Center review board (IRB S2019-1985-0001). Eight patients were involved, consisting of 15 lesions on the upper eyelid and 6 on the lower eyelid. The operations using our new surgical modality were completed with informed consent. Preoperatively, we checked the proportions and dimensions of the lesions and documented their grade according to a previous classification, by defining the location [5] (Table 1). The area proportion was estimated by the count of image pixels on preoperative photography. The surgeries were accomplished by a single surgeon. Preoperative photography and postoperative follow-up photography were taken. The complications consisting of functional and aesthetic outcomes were recorded during the postoperative follow-up period. Patients were asked to measure the degree of satisfaction including conventional possible complications such as flap necrosis, recurrence, lagophthalmos, hypertrophic scar, and general gross appearance. Specific complications that could occur by our new method were also noted. This report adhered to the ethical principles outlined in the Declaration of Helsinki as amended in 2013.

## 3. Surgical Technique

The boundary of the xanthelasma was marked, and the incision for the height of the fold was designed according to the patient's desire while not leaving any lesion on the lower flap. The amount of skin excised additionally for the blepharoplasty incision line on the upper eyelid was based on the degree of the skin laxity and bulkiness.

Local anesthesia using 1% lidocaine with 1 : 100,000 epinephrine was infiltrated. The skin encompassing the xanthelasmas and the orbicularis oculi muscle at the location of the lesion was removed with a no. 15 blade along the schemed line. The incision along the existing eyelid fold was made, for the redistribution of loose redundant skin, to meet the demand of the required fold line.

After the total tissue removal, the orbicularis oculi musculocutaneous flap was widely elevated extending through the whole upper eyelid on the lateral flap by first priority. Medial flap and lateral flap are divided by the excision line of the lesion, expanding apart from the traditional blepharoplasty incision line. In case excessive tension results, after the advancement of the lateral flap to repair the defect, then the medial flap is elevated. If medial flap is used, the range of dissection should contain the lateral border of the nasal root. Special caution is taken as the application of the medial flap can cause an undesirable neo-Mongolian fold. Raising the lateral flap solely to manage the defect is recommended if the tension is not too high.

Fat maneuver is conducted by opening the orbital septum beneath the orbicularis oculi muscle. The fat should be eliminated minimally or preserved since it can serve as a barrier to prevent unnecessary adhesions that usually happen later, under the orbicularis oculi muscle musculocutaneous flap.

**Figure 2:**
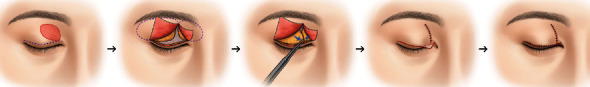
Illustration of surgical technique. (Above) Using only the lateral flap can be enough to cover the defect when the lesion is minimal. (Below) If the following defect is relatively wide, the medial flap could be applied in addition to the lateral flap.

**Figure 3:**
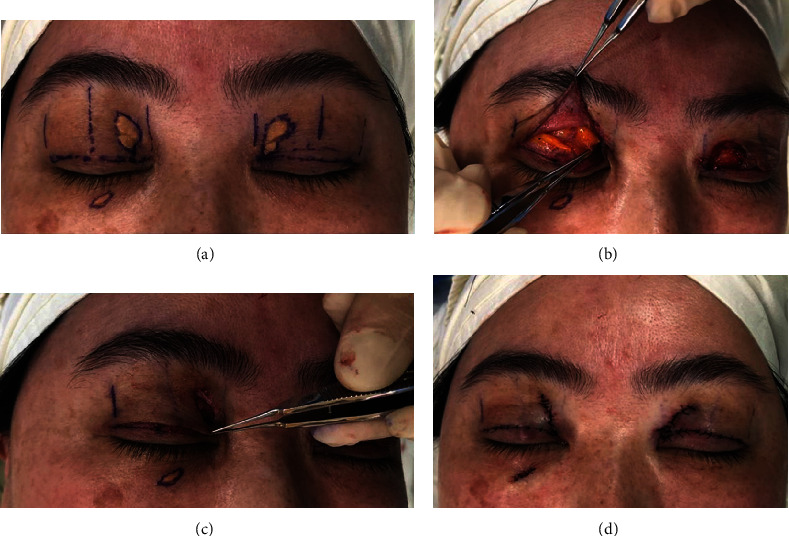
Surgical procedures. (a) Preoperative design. (b) After lesion removal, the orbicularis oculi musculocutaneous flap was widely elevated with fat maneuver. (c) Lateral flap is advanced to the defect distributed by even tension after lower flap with tarsodermal or tarso-dermo-levator fixation. (d) Postoperative photography.

The lower flap is sutured by tarsodermal or tarso-dermo-levator fixation with 7-0 nylon sutures at 3 points to get the desired height of the eyelid fold. Orbicularis oculi muscle musculocutaneous flap is advanced to the medial direction for the lateral flap and to the lateral direction for the medial flap, as per the requirement, to cover the defect. Previously planned additional remnant skin is trimmed according to the tension of flap. Care should be taken when the orbicularis oculi muscle and the skin are closed layer by layer, in order that there is even tension distribution on the upper flap, without creating a tension band. If there is no choice but for the tension band to be created, then the band should be near the fold line, by adjusting the tension of the flap. To avoid a triple fold, the septal fat should be located just below the incision line when the skin is closed. Skin closure was carried out with 6–0 black silk sutures (Figures 2 and 3). On completion of the surgery, taping with a hydrocolloid bandage on the upper eyelid to prevent triple fold formation is essential and should be kept for 2 weeks.

## 4. Results

The mean patient age was 51.5 years (range 42–61 years). The average dimension of excised xanthelasmas lesion was 9.25 mm (horizontal length) × 8.86 mm (vertical height). The average proportion of area was 7.19% with the length proportion of 21.9% horizontally and the height proportion of 39.4% vertically. There were four patients with grade III xanthelasmas, two with grade I, and two with grade II. All the lesions on the upper eyelids were located on the medial aspect with 9 being exterior to the extent of the existing eyelid fold line. The personal data are presented in Table 2.

**Figure 4:**
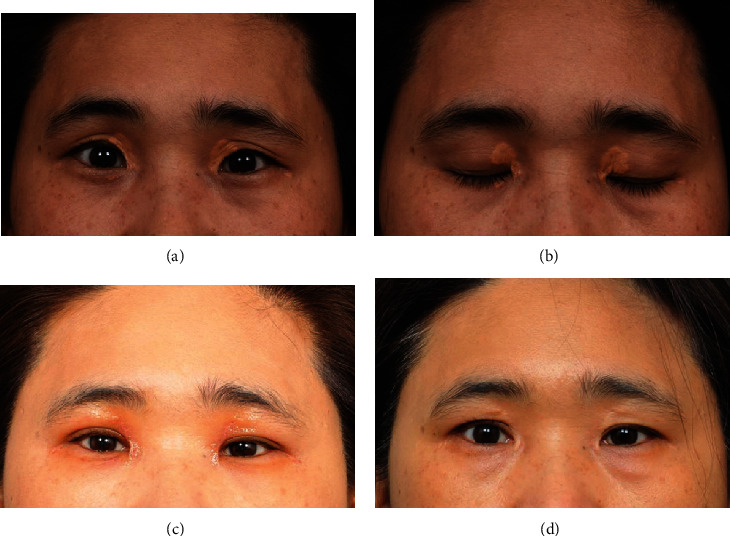
Large defect up to half of the horizontal length can be covered resolving comorbid matters like sunken eyelid, levator function asymmetry, or ptosis by fat reposition and levator manipulation. (a, b) Preoperative photography. (c) Photo at 1 month postoperatively showing neo-Mongolian fold. (d) At 2 months after revision.

**Figure 5:**
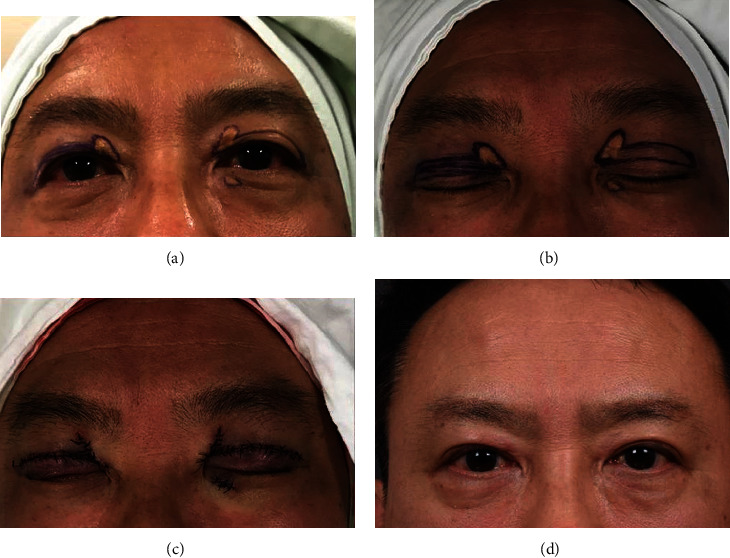
(a–c) In patients with existing double eyelid line, fold height is handled without making a new eyelid fold by drawing the most tensive line to previous line, and puffy lax eyelid is corrected by further resection of the skin along the fold line. (d) Postoperative photo at 5 months.

In all the cases, the wounds were healed, primarily owing to complete flap survival without any complications of wound infection, hematoma, or necrosis. During the 6–19-month follow-up period with an average of 9.8 months, no cases of recurrence, ectropion, lagophthalmos, hypertrophic scar, hypo- or hyperpigmentation, or remarkable deformed morphology like eyelid distortion were noted in any patients. All the patients were satisfied with the overall aesthetic results, and functional outcomes were excellent without any working impairment (Figures 4 and 5). However, complications peculiar to our procedure were demonstrated as one patient had a triple fold with neo-Mongolian fold and another had only a triple fold. Triple fold with neo-Mongolian fold was corrected later, and the patient was gratified finally. Among the others, two trivial dog ear deformities were found.

## 5. Discussion

There have been a lot of treatment methods both local and systemic for xanthelasma treatment. The causative etiology should be confirmed by systemic evaluation first, to correct the underlying disease and control it by drug therapy or lifestyle modification [5]. By following these treatments, recurrence can be diminished.

Despite its benign characteristics, most of the patients favor to remove the lesion due to aesthetic issues of conspicuous location and a tendency of slow enlargement. Local treatment can be achieved by radiofrequency-assisted vaporization of large cosmetically unacceptable lesions [12], cryosurgery [13], medication [14, 15], chemical peeling with trichloroacetic acid [16], laser [17], and surgical excision [11, 18]. The choice of a treatment modality depends on the size and location of the lesion and reflects the patients' preference. Except for surgical excision, other options can be associated with some demerits: longer treatment periods with multiple times of procedure application, probability of relatively high recurrence, and the risk of hypo- or hyperpigmentation changes including persistent erythema and scarring [17]. Simple excision, uncapping [19], microsurgical inverted peeling [6], skin grafting [7], and modified blepharoplasty incision incorporating turnover flap [8], switch flap [9], or hinged flap [10] have been reviewed as surgical treatments.

Excision with primary closure, however, can cause the medial skin to look deficient and aggravate lateral hooding in older patients in a morbid state. Large defects can create too much tension resulting in flap necrosis, hypertrophic scar, and eyelid retraction. Post-skin grafting, the scar contracture might exacerbate not providing appropriate color and texture, complicated with pigmentation problems, and the likelihood of graft failure. Uncapping and peeling have similar pitfalls due to the vulnerability of the flap, with weak perfusion, and the feasibility of an incomplete extraction. Many local flaps also have some drawbacks. Modified rhomboid flap, local advancement flap, and bilobed flap were developed; these flaps were used in combination with skin graft [11]. The switch flap and turn over flap have a bulky appearance due to muscle pedicle movement, thus unfavorable for old patients with puffy eyelids. Besides, these options do not match the eyelid zone as a result of 180° flap rotation, producing an unnatural appearance [8, 9, 20].

Our technique has diverse advantages. Firstly, the use of a flap adjacent to the defect shares a similarity in color and texture with the eyelid zone [20]. It is natural looking as there is no additional donor scar. While the basic concept of using the nearby orbicularis oculi musculocutaneous flap existed, we refined it. The loose upper eyelid skin of post-middle-aged patients can be used in blepharoplasty, to repair the defect. Because the extent of excision for the lesion lies naturally within the upper blepharoplasty marking, a horizontally orientated xanthelasma lesion can be excised during the skin and muscle incision, naturally creating a musculocutaneous flap. In order to excise a lesion placed along a vertical vector and achieve sufficient medial advancement, the muscle needs to be excised in a vertical direction. If the vertical excision demand is within 5 mm and the amount of medial advancement needed is small, the xanthelasma lesion placed as a vertical vector can be corrected by skin excision alone. However, when dealing with larger defects, moving the lateral and medial flap to the horizontal vector makes it possible to cover a large defect, up to half of the horizontal length of the upper eyelid. Undermining widely and rearrangement can also have merits. Extensively elevated flaps can handle a large defect easily owing to the rich blood supply of the eyelid [21]. Even dissecting is not that difficult as the plane is obvious, minimalizing bleeding in the surgical view. If patients have comorbid matters in the process of aging, like a sunken eyelid, entropion, levator function asymmetry, or ptosis with weakened levator aponeurosis, it can also be fixed simultaneously as fat reposition and levator manipulation can be performed in the broad operative field by undermining (Figure 4). The postoperative appearance was also cosmetically acceptable. The creation of a double eyelid was possible for aesthetic purposes when patients with Asian eyelids desired it. With respect to controlling the posture of a double eyelid line as desired, it was possible due to delayed additional skin excision. In patients who already have a double eyelid line, we can simply handle the fold posture by drawing the most tensive line to the previous line, during the closure. Aging eyelid combined with puffy and lax eyelids can be also corrected by further resection of the skin. Designing the excision by making it wider on the lateral side can cause tensive closure to improve the lateral hooding, similar to the effect of subbrow excision (Figure 5).

However, there are several pitfalls in this method. It creates a vertical scar that can be remarkable. While this is inevitable when treating larger lesions, scarring can be minimized through hybrid treatment options. Methods of minimizing the incision line for vertical lesions have been introduced, including a combination of trichloroacetic acid or radiofrequency treatment and surgical excision [22, 23]. The elevation of the medial flap can produce the complication of the neo-Mongolian fold. Moreover, patients can feel somewhat uncomfortable on opening the eye during the first few months due to a tight tension. The illusion of a small eye can be induced without damage or manipulation of the levator palpebral superioris muscle during the operation. This should be warned preoperatively, and epicanthoplasty later on can solve the problem. The most concerned trouble is triple fold formation. To prevent this, high tension point should not be developed, and tension should be distributed evenly through the flap when it is sutured. If it is occurring inevitably, then traction force needs to be drawn to manipulate the fold line as much as possible. Insertion of orbital fat under the skin flap can also be helpful in acting as a barrier during closure. Additionally, taping of the intended fold line should be retained for more than 2 weeks.

The limitations of this study include a small number of cases with a rather short-term follow-up period. To generalize the results, further prospective randomized studies enrolling a wide range of the population with young, old, female, or male patients should be done, to estimate the effect of each specific property in terms of tension and laxity.

In conclusion, excision of xanthelasma usually leads to an extensive defect on the medial side of the upper eyelid. If it is unacceptable, a thorough confident surgical strategy is needed. Hasty treatment without careful preparations can induce unsatisfactory results for both doctor and patient. Our methods had various advantages with satisfactory outcomes. It therefore could be the preferred option for the management of xanthelasma palpebrarum on the upper eyelid.

## Figures and Tables

**Table 1:** Classification of xanthelasma palpebrarum.

	Location and extent of the lesion
I	Only the upper eyelids
II	The medial canthal area
III	The medial side of the upper and lower eyelids
IV	Diffuse involvement of the medial and lateral side of the upper and lower eyelids

**Table 2:** Case summary.

No.	Age (years)/sex	Size left (mm^2^)	Size right (mm^2^)	Grade	Results (functional/aesthetic)	Follow-up (months)	Wound problem	Other complications
1	57/M	12 × 10	10 × 10	III/III	Excellent/satisfactory	12	None	Dog ear deformity
2	44/F	15 × 15	15 × 14	III/III	Excellent/satisfactory	19	None	Triple fold, neo-Mongolian fold
3	61/M	8 × 7	8 × 7	I/I	Excellent/satisfactory	11	None	None
4	57/M	7 × 6	7 × 6	II/II	Excellent/satisfactory	10	None	Dog ear deformity
5	52/M	11 × 5	11 × 4	III/II	Excellent/satisfactory	8	None	None
6	49/F	11 × 8	11 × 7	III/III	Excellent/satisfactory	8	None	Triple fold
7	42/F	8 × 4		I/-	Excellent/satisfactory	6	None	None
8	50/F	10 × 8	7 × 6	III/III	Excellent/satisfactory	5	None	None

## Data Availability

All data generated during the current study are available from the corresponding author on reasonable request.
